# Relationship between balance function and QOL in cancer survivors and healthy subjects

**DOI:** 10.1097/MD.0000000000027822

**Published:** 2021-11-19

**Authors:** Shinichiro Morishita, Ryo Hirabayashi, Atsuhiro Tsubaki, Osamu Aoki, Jack B. Fu, Hideaki Onishi, Tetsuya Tsuji

**Affiliations:** a Department of Physical Therapy, School of Health Sciences, Fukushima Medical University, Fukushima, Japan; b Institute for Human Movement and Medical Sciences, Niigata University of Health and Welfare, Niigata, Japan; c Faculty of Rehabilitation, Shijonawate Gakuen University, Osaka, Japan; d Department of Palliative, Rehabilitation & Integrative Medicine, University of Texas MD Anderson Cancer Center, Houston, TX; e Department of Rehabilitation Medicine, Keio University School of Medicine, Tokyo, Japan.

**Keywords:** balance function, cancer survivors, physical therapy, quality of life, rehabilitation

## Abstract

A previous study reported that cancer survivors exhibit decreased postural stability compared to age-matched controls. Another study showed that cancer survivors have a lower quality of life (QOL) compared to healthy subjects, and there was a significant relationship between muscle strength and QOL in cancer survivors. We aimed to investigate differences in the associations between balance function and QOL in cancer survivors and healthy subjects.

Forty-one cancer survivors and 33 healthy subjects were included. Balance function was evaluated using the timed up and go test, and body sway was tested using a force platform. QOL was assessed using the medical outcome study 36-item short-form health survey.

Cancer survivors exhibited significantly higher timed up and go and lower QOL than that of healthy subjects (*P* < .05). There was a significant association between body sway and QOL (*P* < .05) among cancer survivors. However, healthy subjects had subscales for QOL related to the body sway test parameters more frequently than cancer survivors (*P* < .05).

Cancer survivors’ balance function may have little effect on QOL, unlike in healthy subjects.

## 1 Introduction

A previous study reported that cancer survivors exhibit decreased postural stability compared to age-matched controls.^[1]^ Another previous study reported that cancer survivors have reduced balance function when compared to healthy subjects.^[2]^ Other study showed that cancer survivors have a lower quality of life (QOL) than that of healthy subjects, and there was a significant association between muscle strength and QOL in cancer survivors.^[3]^ Cancer survivors with peripheral neuropathy had decreased balance function and worse QOL scores than those without peripheral neuropathy, particularly in the domain of physical functionality.^[4]^ Thus, balance function may be related to QOL among cancer survivors. We aimed to investigate differences in the associations between balance function and QOL, comparing cancer survivors to healthy subjects.

## 2 Methods

This was a prospective, observational study of balance function and QOL in cancer survivors and healthy subjects. Subjects were recruited between August 2017 and September 2018. Forty-one cancer survivors and 33 healthy subjects were included, all of whom were assessed once. The survivors’ cancer diagnoses included breast (n = 22), colorectal (n = 3), acute leukemia (n = 3), endometrial (n = 2), thyroid (n = 2), lung (n = 1), retroperitoneal sarcoma (n = 1), Ewing sarcoma (n = 1), tongue (n = 1), cervical (n = 1), ovarian (n = 1), bladder (n = 1), testicular (n = 1), and malignant lymphoma (n = 1). The time since the cancer diagnosis of all survivors was >1 year. The Niigata University of Health and Welfare Institutional Committee on Human Research (Approval No. 18065-180820) approved the study. Written informed consent was obtained from all subjects.

### 2.1 Balance function

#### 2.1.1 Timed up and go (TUG) test

Timed up and go (TUG) is a reliable, valid test for quantifying functional mobility.^[5]^ The time taken to complete the test was recorded. Participants performed the TUG twice, and the faster of the 2 measurements was used for analysis.

#### 2.1.2 Body sway testing

Body sway was measured using a gravicorder force platform (GS-10, Anima Inc, Tokyo, Japan) to investigate postural stability among the subjects. Subjects stood for 30 seconds while looking at a 3 cm-diameter round mark placed 2 m away at eye level. The center of pressure (CoP), as the index for postural stability, was measured once using the gravicorder at a 20-Hz sampling rate. Tasks were performed with eyes both opened and closed. The total CoP length (cm), length per area (cm/cm^2^), environmental CoP area (cm^2^), rectangular CoP area (cm^2^), and root mean square (RMS) of the CoP (cm^2^) were calculated.

### 2.2 Health-related QOL

General health (GH)-related QOL was assessed using the medical outcome study 36-item short-form health survey. The 36-item short-form health survey assesses physical and mental health components across 8 domains: physical functioning (PF), physical role function (PR), bodily pain, GH, vitality, social functioning, emotional role functioning, and mental health. This self-administered questionnaire is widely used, particularly among cancer survivors.^[6]^

### 2.3 Statistical analysis

The results are presented as means ± standard deviations. We compared demographic data between cancer survivors and healthy subjects using Student *t* test for continuous variables and Pearson chi-squared test for ordinal variables. Two-tailed unpaired *t* tests were used to compare balance function and QOL between the 2 groups. Pearson *r* was used to evaluate the association between balance function and QOL. Statistical analysis was performed using SPSS 19.0J (SPSS Japan Inc., Tokyo, Japan). *P*-values < .05 were considered statistically significant.

## 3 Results

### 3.1 Clinical and demographic characteristics

No significant difference was observed in the mean age (±standard deviation), male-to-female ratio, mean height, bodyweight, or body mass index between the 2 groups (Table 1).

**Table 1:** Clinical and demographic characteristics and balance function between cancer survivors and healthy subjects.

Characteristics	Cancer survivors (n = 41)	Healthy subjects (n = 33)	*P*-value
Age, yr	49.6 ± 10.5	49.2 ± 11.7	.895
Men, n (%)	8 (20)	9 (27)	.43
Female	33 (80)	24 (73)	
Height, cm	160.2 ± 6.1	161.7 ± 7.9	.378
Body weight, kg	59.3 ± 10.5	56.4 ± 14.6	.317
BMI	23.1 ± 3.9	21.3 ± 4.0	.059
Timed up and go test (s)	5.8 ± 1.1	5.2 ± 0.9	.026
*Eyes open condition*			
Length of CoP (cm)	39.4 ± 14.9	39.2 ± 11.5	.945
Length/environmental area (cm/cm^2^)	40.3 ± 33.5	40.8 ± 24.2	.936
Environmental area of CoP (cm^2^)	1.7 ± 1.4	1.3 ± 0.9	.156
Rectangle area of CoP (cm^2^)	5.7 ± 3.8	4.8 ± 2.7	.244
RMS of CoP (cm^2^)	2.2 ± 1.4	1.7 ± 1.1	.113
*Eyes closed condition*			
Length of CoP (cm)	54.4 ± 22.1	51.1 ± 19.2	.506
Length/environmental area (cm/cm^2^)	38.5 ± 32.0	42.5 ± 22.4	.544
Environmental area of CoP (cm^2^)	2.1 ± 1.6	1.6 ± 1.1	.134
Rectangle area of CoP (cm^2^)	7.8 ± 6.5	6.1 ± 4.0	.191
RMS of CoP (cm^2^)	2.3 ± 1.6	1.9 ± 1.1	.283

Values are presented as means ± SD unless stated otherwise. Statistical testing at baseline was performed using independent Student *t* tests or Pearson χ^2^ tests. BMI = body mass index, BW = body weight, CoP = center of pressure, RMS = root mean square, SD = standard deviation.

### 3.2 Balance tests

TUG time was significantly higher in the cancer survivors than in the healthy subjects (*P* < .05, Table 1). There were no significant differences in body sway test parameters between cancer survivors and healthy subjects (*P* > .05), regardless of the testing conditions (ie, eyes open or closed).

### 3.3 Health-related QOL

PF, PR, and GH were significantly lower in cancer survivors than in healthy subjects (*P* < .01). No significant differences in other subscales were observed between the 2 groups.

### 3.4 Associations between balance function and QOL

When cancer survivors had their eyes open, the length per area, environmental CoP area, rectangular CoP area, and CoP RMS were significantly correlated to PF (*P* < .05, Table 2). Additionally, the length per area was significantly correlated to PR (*P* < .05). However, the other parameters were not significantly associated with the QOL subscales. When healthy subjects had their eyes open, the environmental CoP area and rectangular CoP area were significantly correlated to PF (*P* < .05). Similarly, with their eyes closed, the CoP length and environmental CoP area were significantly correlated to PF (*P* < .05). When healthy subjects had their eyes open, the CoP length, environmental CoP area, rectangular CoP area, and CoP RMS were significantly correlated to GH (*P* < .05). With eyes both opened and closed, the environmental CoP area and rectangular CoP area were significantly correlated to emotional role function and PR (*P* < .05). When healthy subjects had their eyes open, the environmental CoP area was significantly correlated to mental health (*P* < .05).

**Table 2:** Correlations between balance function and quality of life among cancer survivors and healthy subjects.

	Group	Physical functioning	Role-physical	Bodily pain	General health	Vitality	Social functioning	Role-emotional	Mental health
Timed up and go test (s)	Cancer survivors (n = 41)								
	Healthy subjects (n = 33)								
Body sway testing eyes open condition									
Length of CoP (cm)	Cancer survivors (n = 41)								
	Healthy subjects (n = 33)				−0.39^∗^				
Length/environmental area (cm/cm^2^)	Cancer survivors (n = 41)	0.31^∗^	0.32^∗^						
	Healthy subjects (n = 33)								
Environmental area of CoP (cm^2^)	Cancer survivors (n = 41)	−0.44^∗^							
	Healthy subjects (n = 33)	−0.39^∗^	−0.48^∗∗^		−0.36^∗^			−0.44^∗∗^	−0.40^∗^
Rectangle area of CoP (cm^2^)	Cancer survivors (n = 41)	−0.35^∗^							
	Healthy subjects (n = 33)	−0.39^∗^	−0.49^∗∗^		−0.42^∗^			−0.36^∗^	
RMS of CoP (cm^2^)	Cancer survivors (n = 41)	−0.39^∗^							
	Healthy subjects (n = 33)				−0.38^∗^				
Body sway testing eyes closed condition									
Length of CoP (cm)	Cancer survivors (n = 41)								
	Healthy subjects (n = 33)	−0.35^∗^							
Length/environmental area (cm/cm^2^)	Cancer survivors (n = 41)								
	Healthy subjects (n = 33)								
Environmental area of CoP (cm^2^)	Cancer survivors (n = 41)								
	Healthy subjects (n = 33)	−0.38^∗^	−0.50^∗∗^					−0.45^∗∗^	
Rectangle area of CoP (cm^2^)	Cancer survivors (n = 41)								
	Healthy subjects (n = 33)		−0.44^∗∗^					−0.39^∗^	
RMS of CoP (cm^2^)	Cancer survivors (n = 41)								
	Healthy subjects (n = 33)								

Statistical analysis using Pearson correlation coefficient. Only significant correlation coefficients are presented. CoP = center of pressure, RMS = root mean square.

∗ *P* < .05.

∗∗ *P* < .01.

## 4 Discussion

In this study, we showed that cancer survivors had longer TUG times and lower QOL regarding some subscales compared to those of healthy subjects. Furthermore, we found that body sway parameters were significantly correlated to some QOL subscales in both groups. However, healthy subjects had QOL subscales related to the body sway parameters more frequently than cancer survivors. This suggests that there are characteristic differences in balance function and QOL among cancer survivors and healthy subjects.

TUG has been used to assess mobility and requires both static and dynamic balance. Our findings suggest that cancer survivors have decreased mobility balance function when compared to healthy subjects. However, there were no significant differences in the body sway test parameters, which measured postural stability between the 2 groups.^[7,8]^ One study showed that cancer survivors exhibited longer mediolateral RMS and increased CoP velocity than those of age-matched healthy subjects.^[1]^ Another study showed that adult survivors of childhood cancer who were treated with <12 years of chemotherapy had significantly poorer postural control compared to healthy subjects.^[9]^ However, there was no significant difference in postural control between those treated with >12 years of chemotherapy and healthy subjects.^[9]^ To date, few studies have investigated postural stability among cancer survivors, the results of which are not in agreement.

One study investigated the relationship between postural stability and QOL in elderly adults and reported that QOL could be explained by postural sway variables.^[10]^ We initially expected that cancer survivors would have a more prominent relationship between any parameter in the body sway test and QOL than the healthy subjects would. However, cancer survivors had a minimal relationship between some parameters in the body sway test and QOL when compared to healthy subjects. Our study has a limitation. Our study group was relatively small, and was recruited from a single region, therefore reducing the statistical effectiveness of the findings.

## 5 Conclusion

Cancer survivors had significantly increased TUG and decreased QOL; however, there were no significant differences in the body sway tests between the 2 groups. There was an association between the body sway parameters and QOL among cancer survivors; however, these associations were weaker than those observed among healthy subjects. Cancer survivors’ balance function may have little effect on QOL, unlike in healthy subjects. As previously reported,^[3]^ cancer survivors’ QOL tends to rely on muscle strength opposed to balance function. Future studies should focus on a single cancer diagnosis and use a larger sample size for improved analysis.

## Acknowledgments

The authors are grateful to the study participants and physical therapy students for their help in the collection of muscle strength and balance function data.

## Author contributions

**Conceptualization:** Shinichiro Morishita.

**Data curation:** Ryo Hirabayashi, Atsuhiro Tsubaki.

**Formal analysis:** Shinichiro Morishita.

**Funding acquisition:** Shinichiro Morishita.

**Investigation:** Shinichiro Morishita, Ryo Hirabayashi, Atsuhiro Tsubaki, Osamu Aoki.

**Methodology:** Shinichiro Morishita, Osamu Aoki.

**Project administration:** Shinichiro Morishita.

**Supervision:** Shinichiro Morishita, Tetsuya Tsuji.

**Validation:** Hideaki Onishi.

**Visualization:** Hideaki Onishi.

**Writing – original draft:** Shinichiro Morishita, Jack B. Fu, Tetsuya Tsuji.

**Writing – review & editing:** Shinichiro Morishita, Jack B. Fu, Tetsuya Tsuji.
